# Case of Meningitis

**Published:** 1840-04-11

**Authors:** Edward Hallowell


### CASE OF MENINGITIS.

BY EDWARD HALLOWELL, M. D.

*Read April 6th*, 1840.

Henry T——, æt. two and a half, light hair and eyes, enjoyed good health until the summer of 1839, when he had an attack of cholera infantum of several weeks' duration. He had also a slight diarrhœa in the winter following; with these exceptions, and an occasional cold, never lasting more than a day or two, his health continued uniformly good until Friday, March 13, when he complained of pain in the knee joint; it soon passed off, however, and did not return until the Friday following, when on coming down stairs he complained again of his knee, and said he could not walk. Dr. Pierce saw him in the evening, when he had fever with some difficulty of breathing, for which he had him bled ; in the night he was delirious, and the next morning at ten, was seized with a convulsion. He was again bled and directed powders of calomel, jalap and aloes, every two hours, with stimulating injections ; between half past ten and two o'clock, he had two other attacks equally severe ; blisters were directed to the calves of the legs, and one was applied to the back of the neck.

Dr. P. now requested me to see the case with him, which I did at half past three, when the following symptoms were noticed : decubitus dorsal, body largely developed ; lids of right eye about a line apart, of left three lines; pupils greatly dilated, contracting slowly on exposure to the light ; right arm at times much in motion, the fingers occasionally touching the head, and angles of the mouth ; slight blueness of lips, face livid, pulse one hundred and fifty, small and feeble, not intermittent ; respiration sixty, high, no cough ; skin cool ; bowels costive; been opened three times to-day, by calomel powders, and injections ; abdomen distended and tympanitic, appears painful on pressure ; left knee joint and thigh of same side much swollen. He died an hour after the visit.

*Autopsy, March 24th, twenty-three hours after death.*

*Exterior.*—Embonpoint considerable ; some rigidity of left knee and hip-joint, other joints supple ; left knee and thigh one inch greater in circumference than the corresponding parts of opposite side.

*Head.*—But little blood exterior to the dura mater ; arachnoid pale and perfectly transparent ; a very small quantity of limpid serosity in several of the anfractuosities ; veins of pia mater distended ; the pia mater itself highly injected ; the injection is of a bright red colour, and is not confined to the larger vessels, but extends to their smaller ramifications ; it is most marked at the base of the brain, and upon the convex surface of the middle and posterior lobes ; cortical substance pale ash colour, about two lines in depth ; medullary substance injected, and softened throughout ; no serosity in cavity of ventricles ; the substance of the cerebellum is also softer than in the healthy state.

*Thorax.*—Pleuræ moist, not adherent ; lungs of a deep violet colour externally ; tissue highly engorged, a considerable quantity of dark coloured blood exuding on pressure.

In the apex of the lower lobe of left lung, are a number of crude tubercles of a light yellow colour, and cheese-like consistence ; the surrounding tissue is indurated, and of a grayish red colour ; the indurated portion does not occupy more than an inch in extent; three or four lines below this is another tubercular deposit, four lines in length by about three in breadth, the tissue surrounding which is simply engorged, presenting not the slightest trace of induration. Mucous membrane of trachea and bronchi for the most part pale, presenting a few dotted points on its surface ; consistence normal ; bronchial glands not enlarged or tuberculous. Heart of normal size, two and a half inches in length, measured from origin of aorta upon its anterior face ; circumference at base five inches ; the coronary veins and their ramifications are much congested, the latter presenting fine arborizations of a pale indigo color ; the right ostium venosum is occupied by a large coagulum, the greater part of which consists of fibrine, the rest of dark coloured blood ; they adhere firmly to each other; the fibrinous portion extends into the right ventricle, to the sides of which it is attached. There is little or no blood in the left cavities ; walls of left ventricle three and a half lines in thickness, exclusive of columnæ carneæ ; of right, a line and a half. Valves and lining membrane healthy ; pericardium pale, containing no serosity.

*Abdomen.*—About half an inch of fat beneath the integuments; peritoneum pale and moist; no effusion within its cavity; intestines moderately distended, of a pale yellow colour; liver of natural size, extending about an inch below the ribs ; convex surface marbled with pale yellow and violet ; anterior half of under surface of a tea-green colour, the rest pale yellow ; tissue firm, containing but little blood ; on examining it closely, the acini are observed to be very distinct ; they are of a pale yellow colour ; the surrounding tissue is reddish violet, the two substances being quite distinct ; gall bladder distended with thin dark coloured bile, staining the finger a deep orange. Mucous membrane of œsophagus somewhat congested, a number of small bluish veins being seen upon its surface. Stomach moderately distended, containing a quantity of greenish looking matter without perceptible odour ; its inner surface is coated with a thick layer of ropy mucus, on removing which, the follicles are observed to be in a state of unusual development; they are by far more abundant at the pyloric extremity, a few isolated ones only being seen in other portions of the stomach; in the former situation they are thickly agglomerated, many of them being in juxtaposition; they vary in size from that of a grain of mustard to a line or more in diameter; they present no signs of inflammation, their colour being the same as that of the surrounding membrane, above which they are more or less elevated; in each of them is observed a minute central depression; mucous membrane of a pale onion tint, and of natural consistence, yielding strips one inch and a quarter in length along lesser curvature, six lines in greater, and of four lines in great cul de sac; the small intestine contains a moderate quantity of matter of an orange tint in duodenum, and of a lighter colour in the remaining part of the intestine; the mucous membrane is pale, and of normal consistence, yielding strips five lines in length; the mucous follicles throughout its entire tract are greatly developed, being elevated considerably above the surrounding surface of the intestine; they are about a line in diameter; their central orifice is slightly dilated; the large intestine contains a considerable quantity of fœces of a pale whitish yellow colour; the mucous membrane is perfectly pale, and of natural consistence, yielding strips of about eight lines in length; the surface throughout is studded with mucous follicles in a state of development, each with a greenish point in its centre; they vary considerably in size, the largest being rather more than a line in diameter; the orifices are more or less dilated. No ulcerations are observed in any part of either the small or large intestine. Mesenteric glands somewhat enlarged; the largest about ten lines in length, pale, not tuberculous. *Kidneys* three and a quarter inches in length by two in circumference; cortical substance pale ash colour; tubular reddish violet; consistence normal. *Spleen* four and a half inches in length by two and a half in breadth, of a slate colour externally, rather softer than natural. *Bladder* moderately distended with urine of a pale citron colour; lining membrane healthy.

*Remarks.*—By a great oversight the knee joint was not examined after death, and the symptoms during life were not very carefully noted; there can be little doubt, however, that the joint in question was in a state of inflammation; rheumatism, the only disease with which it could well be confounded, being of rare occurrence in children of that age; taking this for granted, it illustrates the sympathetic relation which exists between serous membranes, in different and even remote parts of the body; it is indeed, not improbable that meningitis more often occurs in connexion with inflammatory affections of the joints in children, than is generally supposed. A case resembling the above, came under my notice in the summer of 1839: it was that of a child five years of age, whose health had been good until about four months before her death, when she received an injury of the ankle joint, from the fall of a heavy body upon it. The joint continued swollen and painful, but notwithstanding this, she was suffered to run about, and caries was the consequence. The limb was kept in a fracture box for several months, with a view of arresting the inflammation and inducing anchylosis, but matter continued to form, and made its appearance at intervals by two openings, one upon the outer, the other upon the inner aspect of the joint. A severe attack of ophthalmia occurred during the treatment, which came near destroying vision, but from this, she entirely recovered. The illness which terminated her life, commenced on the 28th of June. I did not see her until Saturday, the 30th; she had then some fever, but the pulse was soft and compressible; there were exacerbations in the middle of the day and towards evening; the stomach was very irritable, rejecting almost every thing taken into it, and she complained of pain in the head; the two most striking symptoms were the vomiting and the pain in the head, which was constant; it was increased by sitting up in bed; the countenance was rather pale, except during the febrile exacerbations; the tongue was clean, the abdomen supple, respiration natural; the bowels were costive. On the morning of her death she appeared rather better; she sat up in bed and conversed cheerfully; the pulse, however, was accelerated and somewhat tense, and the face rather flushed; at half past one, two hours after visit, she was seized with convulsions, which were of extreme violence, and died the same evening, at 7 P. M.

*Autopsy, June 5th, twenty-four hours after death.*

*Head.*—A considerable quantity of blood, exterior to dura mater; veins of pia mater moderately distended; arachnoid moist and transparent; no effusion beneath; pia mater very minutely injected throughout its whole extent, the injection of a bright red colour, occupying the smaller vessels and their ramifications; cortical substance pale, medullary dotted with blood; the whole substance of the brain softened.

*Thorax.*—Pleuræ pale and moist; lungs slightly engorged, crepitant; no tubercles.

*Abdomen.*—Peritoneum healthy. *Liver* not enlarged, of a chocolate colour, firm; mucous membrane of stomach covered with a quantity of yellowish mucus, readily removed with the handle of the scalpel; surface pale, except at great cul de sac, where it is somewhat injected; mucous membrane of large and small intestine pale, and apparently of normal consistence; the isolated follicles of the large intestine are quite apparent on holding it up to the light, but do not appear to be unusually developed.

*Kidneys* healthy. *Spleen*, *Bladder* and other organs not examined. We have here the case of a child, labouring under a chronic inflammation of one of the joints, attacked with fever of apparently a remittent type; there exists, however, extreme irritability of the stomach with constant pain in the head, both symptoms of severe cerebral disturbance, and which demand, especially in children, the closest vigilance on the part of the physician. Death ensues on the fifth day from the commencement of the febrile symptons, and in about six hours after the occurrence of severe convulsions. The autopsy presents the anatomical signs of acute meningitis without effusion. M. Foville, in alluding to the sympathetic relation which exists between inflammation of the meninges and that of other serous membranes, mentions a case in which it was displayed at a much later period of life. A man sixty years of age was operated upon for hydrocele, by injection; almost immediately after the operation, he was seized with acute inflammation of almost all the synovial membranes, including those of the temporo-maxillary articulations, of the last phalanges of the fingers, and even of the atlas with the odontoid process; anchylosis occurred in several of the joints. All the rational signs of acute meningitis were observed at the same time as those of the inflammation of the joints.*

The first of the cases I have detailed, presents other points of interest; the tubercular deposit is remarkable from its situation, and its limited extent, being found in no other portion of the lungs than the upper part of the lower lobe of the left lung, and in no other part of the body; it occurred too in a child, in the enjoyment apparently of robust health; at first sight it might be supposed that it had followed an attack of lobular pneumonia, and that the tuberculous deposit was the result of a chronic induration of the lungs, but this opinion would seem to be disproved by the previous history, and the appearances after death; the child never had more than a slight cold, lasting but a day or two, and no marked signs of bronchitis were observed at the autopsy; at some distance below the indurated portion, another tuberculous deposit is observed in a part of the lung, which up to the day of the child's death must have been perfectly healthy, being only in a state of very recent engorgement; a fact which proves, we think, that the induration was the consequence and not the cause of the tuberculous deposit contained within it. That tubercles are of inflammatory origin is maintained by the best authority, but in by far the greater number of cases, we are disposed to think them quite independent of such a process, being the effect of depressing causes acting upon the whole system; such was probably the case in the present instance.—The parents are quite poor, living in a small house in a narrow, illy ventilated street, and the father is of intemperate habits. The case is also interesting, from the extreme development of the mucous follicles of the stomach and intestines. Even in cholera infantum, and in advanced stages of the tuberculous disease, in children, it is rare to find them occupying so great an extent of surface. It will also be remembered, that there was no corresponding diarrhœa.

* Dict. de Medecine Pratique. Art. Meningite.

